# Skene's glands abscess an overlooked diagnosis in acute lower urinary symptoms

**DOI:** 10.1016/j.radcr.2021.09.006

**Published:** 2021-10-02

**Authors:** Stefania Tamburrini, Carmine Vascone, Valeria Marrone, Marco Catalano, Dario Del Biondo, Luigi Gallo, Pasquale Quassone, Marina Lugarà, Maria Gabriella Coppola, Fiore De Simone, Giorgio Napodano

**Affiliations:** a Department of Radiology, Ospedale del Mare-ASL NA1 Centro, Via Enrico Russo, 80147 Naples, Italy; b Department of Obstretics and Gynecology, Ospedale del Mare-ASL NA1 Centro, Naples, Italy; c Department of Urology, Ospedale del Mare-ASL NA1 Centro, Naples, Italy; d Department of Radiology "Università degli Studi della Campania Luigi Vanvitelli", Naples, Italy; e Department of Internal Medicine, Ospedale del Mare-ASL NA1 Centro, Naples, Italy

**Keywords:** Skene's glands abscess, Skenitis, Paraurethral abscess, Lower urinary tract symptoms, ULTRASOUND, US, Magnetic Resonance Imaging, MRI, paraurethral glands

## Abstract

Skenitis refers to the infection of the Skene's glands. Skene's glands are paraurethral glands localized on the upper wall of the vagina. The diagnosis of Skene's glands abscess or infection is usually made based on the history and physical examination, but half of women with para-urethral gland symptoms present with non-palpable lesions and necessitate further evaluation with imaging. Patients may present with chronic urethral pain, recurrent urinary tract infections, unexplained dyspareunia, and dysuria. At imaging Skene's glands are typically located on the anterior vaginal wall, at symphysis level and paramedian to urethra. Clinicians should consider Skenitis in the differential diagnosis of lower urinary tract symptoms. We report a case of a 48-year-old woman with acute lower urinary tract symptoms with a final diagnosis of Skene's glands abscess.

## Introduction

Skene's glands are paraurethral glands localized on the upper wall of the vagina; infectious process of Skene's glands (Skenitis) can cause the obstruction of paraurethral ducts [1], [2], [3], [4]. Symptomatic abnormalities of the female urethra are mostly benign [3], [4], [5], [6], [7] and although urethral diverticula represent the more frequent abnormality, Skenitis should be considered in the differential diagnosis. We discuss a case 48-year-old woman with acute lower urinary symptom and a final diagnosis of non-palpable Skene's glands abscess.

## Case report

A 48-year-old woman referred at our hospital complaining urgency dysuria, dyspareunia, and discharge. She was under antibiotic therapy for the last 5 days (ciprofloxacin 500×2) with no relief of symptoms, urinalysis was negative. On examination, she was febrile with lower abdominal tenderness.

**Fig. 1 (A-D):**
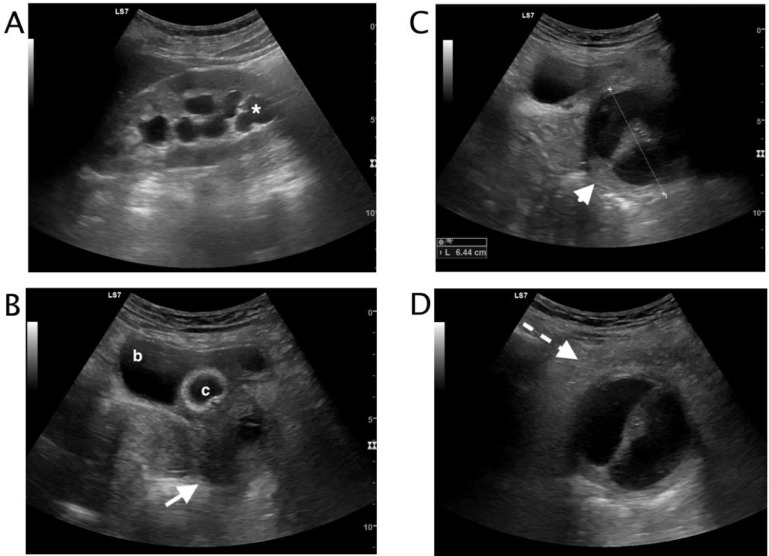
Transabdominal ultrasound (A-D) A. Mildly dilated calico-pelvic system of the left kidney (white asterix). (B) Catheterized bladder (c). Below the bladder (b) an inhomogeneous fluid filled mass was appreciable (white arrow). (C) The fluid filled mass content was slightly inhomogeneous, the mass was septate (white arrowhead) with thick wall and localized below the bladder (maximum diameter 6.44 cm). (D) Fluid perilesional suffusion and fat inhomogeneity were appreciable (dashed arrow) , a hyperechoic spot was visualized in the middle center of the septum.

**Fig. 2:**
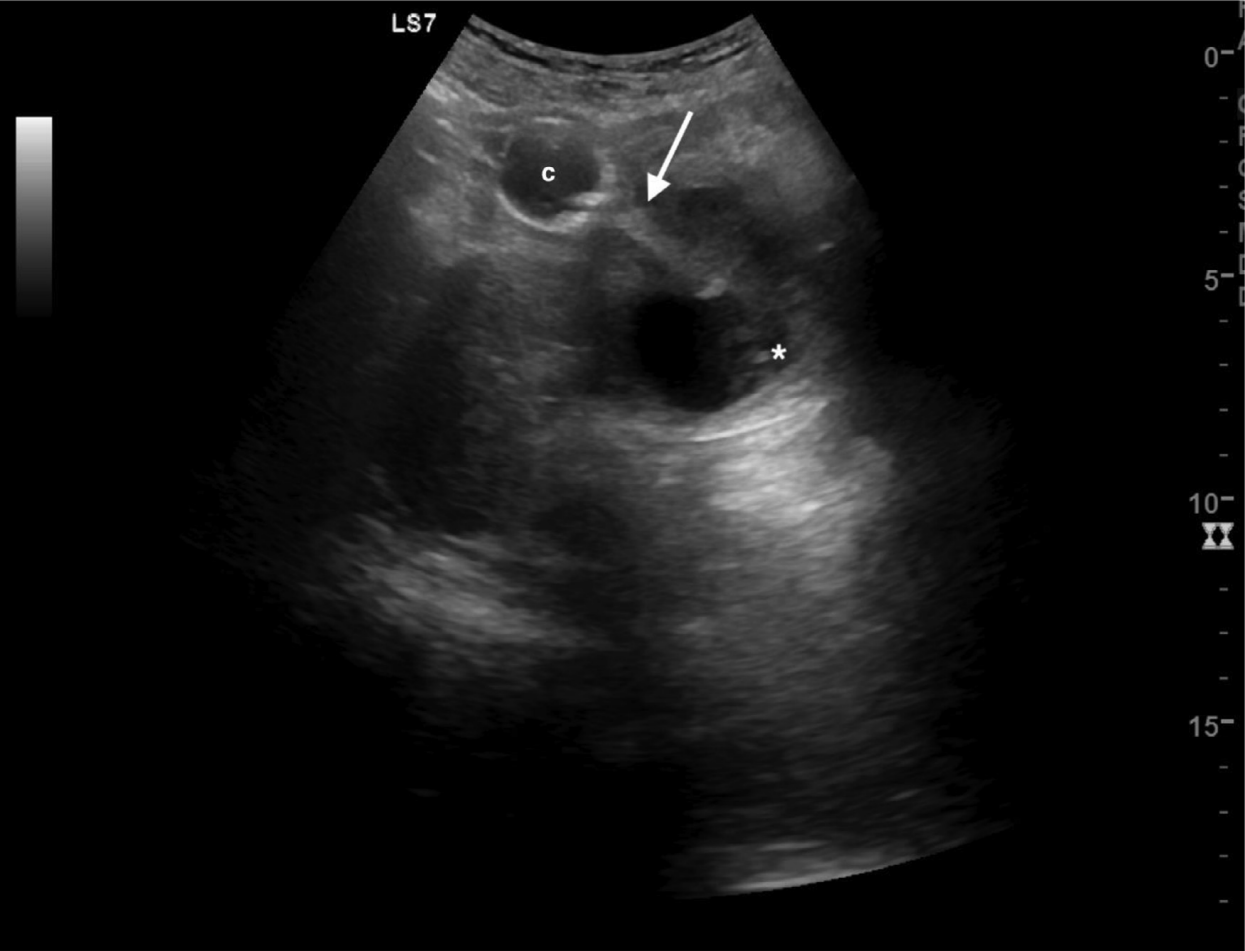
Transabdominal ultrasound after emptying the bladder. The catheter balloon (c) was visible on the upper pole of the lesion, the hyperechoic catheter line run within the midline septum (white arrow). The fluid content is inhomogeneous (white asterix).

Laboratory data included hemoglobin 13,2g/dl (normal value 11.5-17.5), hematocrit 39.9 % (normal value 36.0-55.0), white blood cell count 13,2 10^3^/mm^3^ (normal value 4.2-10.5), neutrophils 84,9% (normal value 40-75), creatinine 0,54 mg/dl (normal value 0.40-0.95), C reactive protein 12,64 mg/dl (normal value 0.0-0.5). A Foley catheter was inserted into the bladder to drain urine. Catheter was clamped and transabdominal ultrasound was performed. Left kidney calico-pelvic mild dilatation was detected (Fig.1 A). During pelvic ultrasound examination, the patient referred intense pain. The bladder was mildly distended (Fig.1 B) , and a fluid filled mass with a thick central septum was visualized (Fig.1 C). The fluid mass content was slightly inhomogeneous, the fluid mass presented thick walls and the perilesional fat was inhomogeneous , the lesion was placed inferiorly to the bladder (Fig.1 D)After emptying the bladder, the catheter seemed to pass through the middle septum (Fig. 2). An ultrasound diagnosis of pelvic fluid filled mass with left kidney urinary stasis was formulated. Gynecological examination was performed. At clinical examination, the patient had cervical motion tenderness on bimanual vaginal examination with some adnexal tenderness.

**Fig. 3 (A-B):**
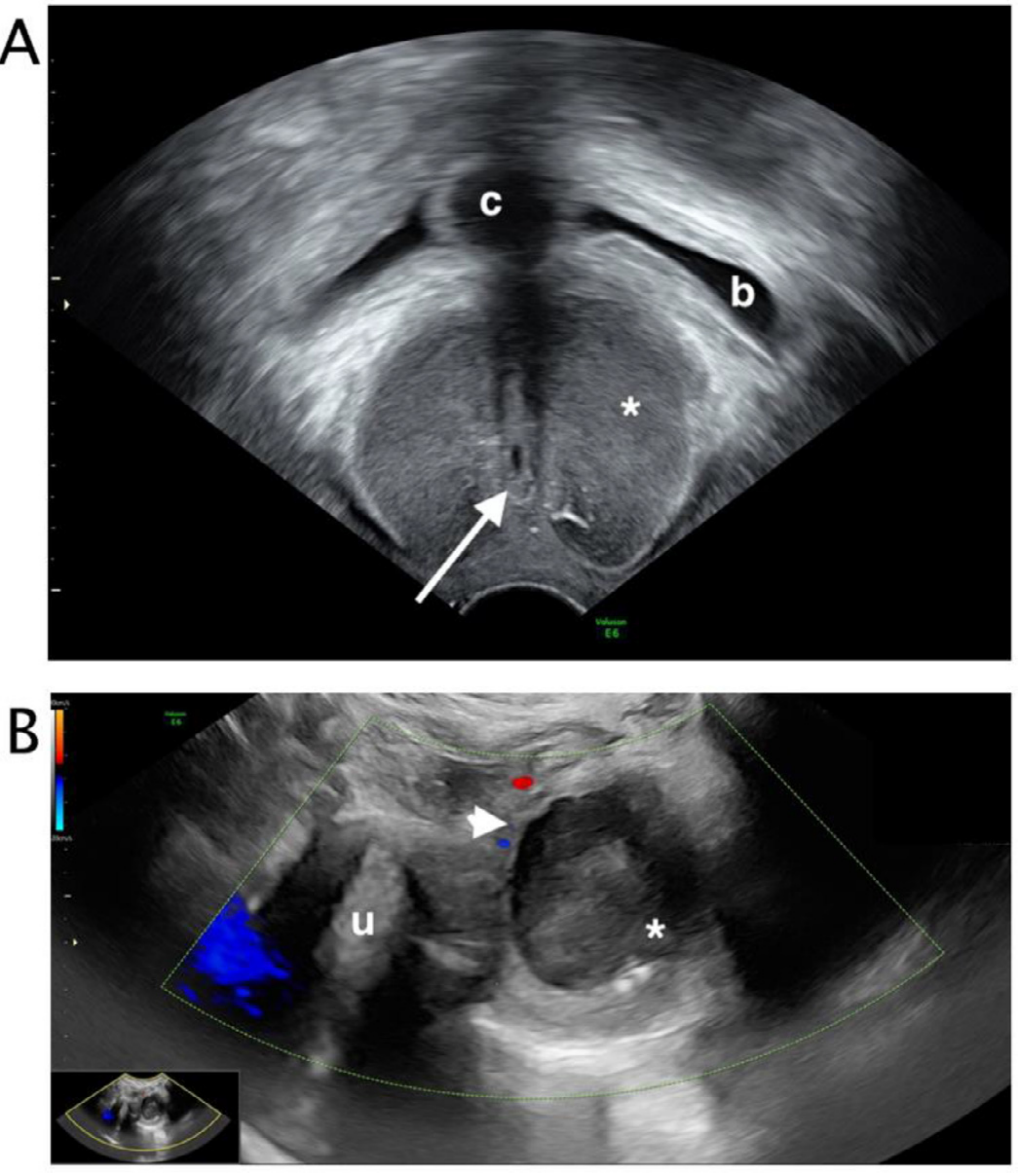
Transvaginal ultrasound. At transvaginal ultrasound, a bilocular cyst with a non-vascularized septum (white arrowhead) (B), low level content and regular walls (white asterix), was visualized below the catheterized (c) bladder (b) and anterior lateral to the urethra (white arrow) (A). There was no communication between the cyst and urethra. Uterus (u).

At transvaginal ultrasound, a bilocular cyst with a non-vascularized septum, low level content and regular walls, below the bladder and anterior lateral to the urethra was visualized. There was no communication between the cyst and urethra (Fig. 3).

**Fig. 4:**
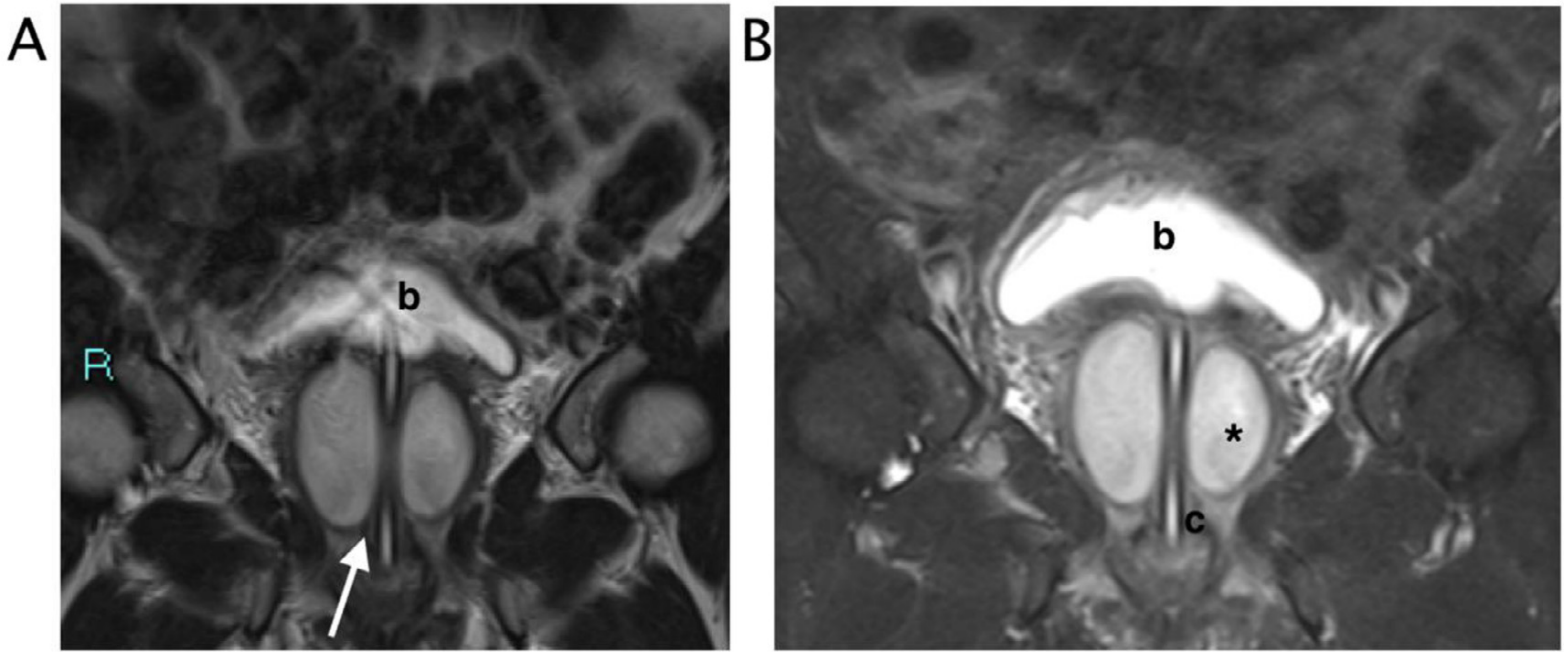
MRI. The bilocular fluid collection is located just laterally to the external urethral meatus and inferior to the pubic symphysis. (A) Coronal T2 Turbo-spin-echo sequence with selective fat suppression. (B) Coronal T2 Turbo-spin-echo sequence. The fluid filled collection with internal mildly heterogenous hyperintense signal (black asterix) is localized laterally to the urethra (white arrow), the catheter line (c) run in the middle. Bladder (b).

**Fig. 5 (A):**
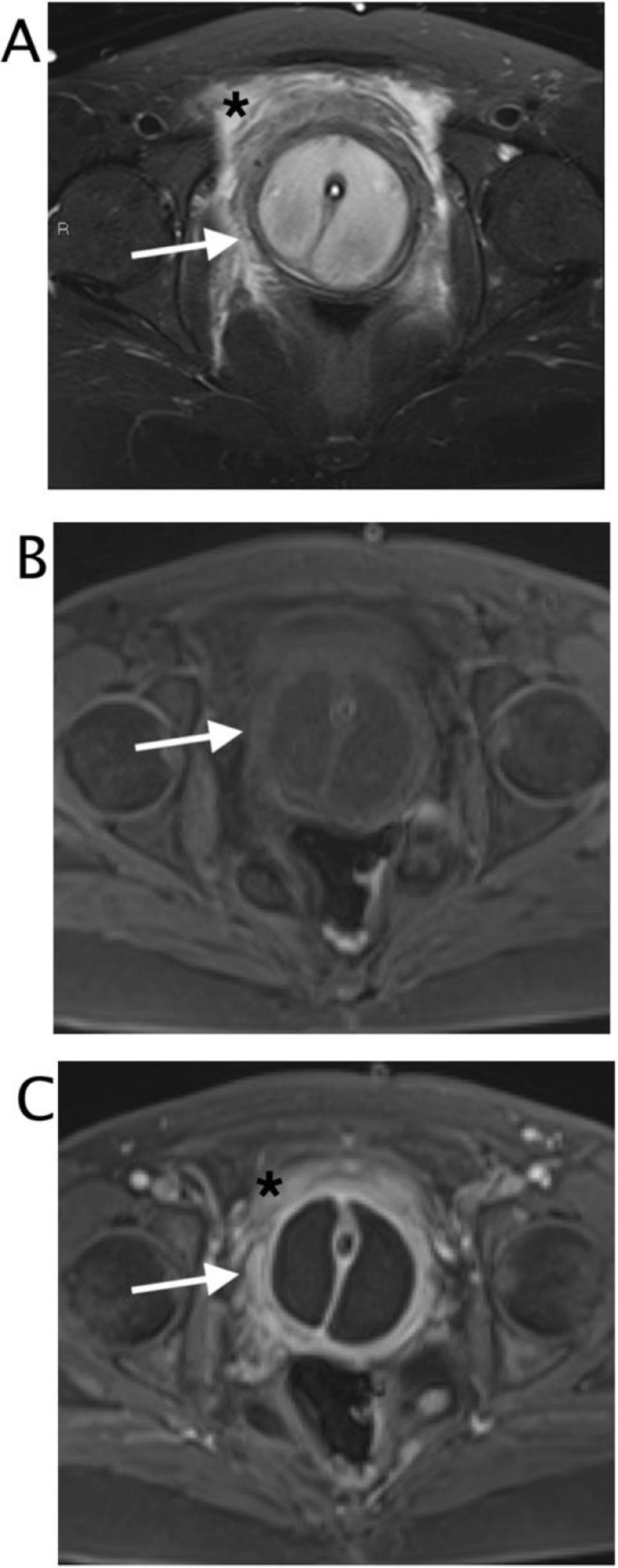
Axial T2 Turbo-spin-echo sequence with selective fat suppression. The inhomogeneous bilocular fluid collection is localized around the urethra. Intense peripheral swelling was appreciable (black asterix). (B) Axial T1 vibe with selective fat suppression. The round image presented thick walls (white arrow), internal isointense to muscle signal. (C) Axial T1 vibe with selective fat suppression after contrast in venous phase, The walls appeared thickened and smooth (white arrow)

The patient was admitted to the hospital and a pelvic Magnetic Resonance Imaging (MRI) with contrast was performed. The fluid filled mass was appreciable lateral to the external urethral meatus and inferior to the pubic symphysis. At MR imaging, the lesion was oval, hyperintense on T2-weighted images. The walls were thick and there was perilesional fat stranding. The infectious was suspected on the basis of lower T2 signal, higher T1 signal and wall enhancement (Fig. 4 and 5). A final diagnosis of infected paraurethral cystic mass was formulated.

At surgery, the abscess was treated with the technique of marsupialization. A vertical incision was made on the anterior vaginal wall, the abscess drained spontaneously, the cavity was copiously irrigated with betadine. Sutures were positioned to evert the cyst wall. The cultural exam revealed infection sustained by Escherichia Coli and Staphyloccoccus Haemolyticus.

## Discussion

The normal female urethra is 3–4 cm in length, suspended to the pelvic sidewall and “pelvic fascia” by the urethropelvic ligament, which is composed of two layers of fused fascia. Masses that present adjacent to the urethra or at the meatus between the “fascia” layers, arise from the vaginal wall,or the urethra itself [3]. Skene's glands were firstly described by Reinier de Graf and sequent named by Alexander Skene [8]. The skene's glands are paraurethral glands that arise from the urogenital sinus, they are located inferiorly and laterally on either side of urethra, adjacent to urethral meatus in the vestibule [9]. The main ducts draining the gland open on either side of the urethra, in the posterolateral position, and are lined by transitional type epithelium, which merges with the squamous epithelium of the vestibule [1]. They are considered the female homologues of the prostate gland [10, 11], they are present in two-thirds of women [12] and seem to be responsible for female ejaculation [11, 13]. Like the male prostate, the Skene's glands can develop adenomas, which are benign glandular tumors, or adenocarcinomas, which are malignant glandular tumors, however, cancer of the Skene's glands is less common than that of the prostate. Bacterial infections can lead to obstruction of paraurethral ducts and determine the formation of cyst or abscess. When infected, the Skene's gland becomes enlarged and tender, a condition known as skenitis, and repeated infections may lead to obstruction of the gland and result in a suburethral cyst or an abscess cavity. Large Skene's gland cyst or abscess may cause urethral obstruction and urinary retention [14].The frequency of Skene duct cyst may be higher and overlooked than the number of reported cases, they can be incidentally diagnosed during evaluation of women with refractory lower urinary tract symptoms or during dissection (or palpation) at the time of anti-incontinence surgery [5]. Skene abscesses are most frequent in the third to fourth de-cade of life, although a case occurring children has been reported [1]. Half of Skene's gland abscess can be palpable, and the diagnosis is based on the history and physical examination. At clinical evaluation the anterior vaginal wall and area long the urethra should be inspected and palpated for any nodules, masses or point of tenderness, palpation of the bladder is also essential to elicit bladder tenderness [15]. Clinical assessment of women with para-urethral gland symptoms and non-palpable lesions may be difficult, necessitating further evaluation with imaging [5, 16]. Patients that present with chronic urethral pain, recurrent urinary tract infections, or unexplained dyspareunia, dysuria can be a diagnostic challenge. In these patients the diagnosis of Skene's gland cyst or abscess should be considered [16]. Dyspareunia and dysuria are the most common presenting symptoms [7, 16, 17]. Although imaging (ultrasound, transvaginal ultrasound, and MRI) cannot distinguish benign from malignant lesion, clinicians should consider Skene's glands anomalies in the differential diagnosis. Clinicians should be aware of the characteristically Skene cystor abscess appearance: they are typically located at the anterior vaginal wall, at symphysis level and paramedian to urethra. At Emergency Ultrasound evaluation of female patient with urinary tract symptoms, the detection of a fluid paraurethral mass, homogeneous in case cysts or inhomogeneous in case of abscess, should alert the clinician to consider this diagnosis [2, 18]. In case of female perineal cystic lesion, location is the key [19]. Nowadays, voiding cystourethrogram and double-balloon urethrogram are rarely performed [20], instead MRI is noted to have high specificity at 83% and high sensitivity at 100%, along with high positive predictive and negative predictive values at 92% and 100%, respectively Preoperative MRI imaging is used in challenging cases for urethral diverticula [4, 11, [21], [22], [23], [24]]. The differential diagnosis for a vaginal mass is wide and include infection, malignancy, urethral prolapse, urethral diverticulum, ectopic ureterocele, Bartholin cyst, Gartner's duct cyst or abscess [9]. In the case of an infected Skene's gland, conservative therapy with antibiotics is the first-line therapy [16]. However, antibiotics are less effective in larger abscesses and surgical intervention ultimately is the most effective approach if antibiotic therapy fails [4, 17, 23, 24]. In addition, an untreated infected Skene's gland abscess may lead to the development of a urethral diverticulum [3, 16]. There is no clear consensus on the standard surgical management of Skene's gland cysts, it includes cyst removal, marsupialization, puncture and aspiration . Spontaneous drainage has also been reported [9, 17, 25]. In the case reported the final diagnosis was surgical, this may relate firstly to the rarity of the disease, secondarily to the fact that these pathologies are not considered in general emergency settings but are usually diagnosed and treated in specialized context. The characteristic appearance at ultrasound and MRI can help clinicians and radiologists to rapidly diagnosis this kind of disease.

## Conclusions

In female patients with lower urinary tract symptoms, S S Skene's glands abscess and cyst should be included in the differential diagnosis. Although rarely reported, the disease can be underestimated. Characteristically imaging appearance and location can help to rule out this diagnosis.

## Patient consent

Patient's consent not required as patient's identity is not disclosed or compromised.
